# Editors and Editing

**Published:** 1885-12

**Authors:** 


### EDITORS AND EDITING.

It is a compliment when men like Cushing and others, who have written us approvingly, gravely consider the matters presented in the editorial of our October number, entitled "Editorial Responsibilities." In his article in this number, Dr. Cushing suggests ideas that might result in a better literature, if correspondents were imbued with the spirit which actuates him, and if the editor were the ideal being who would alone be competent to put them in practice. But the average writer for the professional journals, all unpracticed though he may be, is exceedingly tender of the children of his brain; the greater their faults the higher do they usually stand in his estimation. Parents are said always to best love those of their offspring who are most deformed. The mere correction of a grammatical error, the striking out of a tautological word, or the rearrangement of a sentence that it may more clearly express the supposed meaning, is apt to be met with an indignant remonstrance, and the intimation that another opportunity for the taking of such liberties will not be afforded the offending editor. He will likely be told that, instead of improving the reading, he has totally misapprehended the idea of the author, and made a muddle of the whole business—as quite possibly he has—for who can fathom the meaning of a writer when he is not quite clear himself as to what he desires to say.

The prevalent belief among correspondents is that an editor must either accept and print an article precisely as received, or reject it altogether, irrespective of the fact that an unpracticed writer may present thoughts of great magnitude, which the editor desires to use, but which are ill clothed. If the latter strikes out some bold flight of fancy, which is quite irrelevant and positively weakening to the argument, it is certain to be the very pet paragraph of the author.

If the editor correct a mere question of fact, he will be taken to task as imposing his own opinions, and declining to allow a fair inquiry into the truth of generally accepted theories. And there is much of justice in the reproof. Had Harvey first proclaimed the circulation of the blood in a professional journal, would the editor have been justified in correcting his assertion, on the ground that the theory was in direct antagonism to accepted facts? Or shall the manuscript of Miller, or Bödecker, or Abbott, be subjected to editorial revision because they are announcing something new, and preaching doctrines that have not as yet received the stamp of true orthodoxy?

What man is wise enough to determine when he may not be crucifying divine truth?

If the editor interlards the communications of his correspondents with interjections of his own, with contradictions and adverse comments, is he not palpably interfering with the right of the author to a fair field? If he accords to the writer the privilege of expressing himself at all, shall he not give him the opportunity to have his say out, without interrupting and breaking the thread of his argument with impertinent oppositions and gainsaying interjections? Does Dr. Cushing like to hold an argument with an adversary who interrupts every third word with an intrusive denial? Is it not the part of good breeding respectfully to listen till the other party has had his say, and then proceed to utterly demolish him? But the editor is supposed, in his own journal, to speak louder than other men. He can always have the last word if he will. How will correspondents relish the eternal snubbing of those interlarded comments? Or with what stomach will they accept the public reproof, the dogmatical pulverizing which an editorial contradiction in a separate article implies? No! the editor must treat his correspondents gingerly, if he would have their continued co-operation.

Then there is another point of view. No doubt editors are the most wise, the most sagacious, the most erudite of men. But there is positively a limit to their learning. It is quite within the bounds of possibility that there are those among the laymen who could put new wrinkles on their horns ; who could teach them the very rudiments of some special branches of knowledge. Shall the editor assume to sit in judgment upon the declarations of those who were intimate with truth before he was even born in science ? The average editor is a man of unbounded gall, but he usually has some of the remnants of modesty left. And if he be not possessed of a spark of humility, he sometimes has a modicum of prudence. Not always, for we have in our mind's eye two or three instances in which an editor beautifully committed himself by an attempted correction of a correspondent who subsequently proved that he knew what he was talking about, while the editor had not the least conception of the real scientific aspect of the case. No, no. There are correspondents of the INDEPENDENT PRACTITIONER at whose feet the editor very gladly sits, that he may draw instruction from those who are his acknowledged masters.

But when there is such a glaring instance of the violation of good taste as is pointed out to us, in a foreign journal, by a correspondent in far away Madrid, there is no question about the editor's responsibility. He has no right to allow a correspondent to make a consummate ass of himself, even though his ears be extraordinarily long. When the braying becomes too conspicuous, the ass should be summarily squelched.

Every editor desires to make of his journal the very best one published. The most of them succeed—at least to their own satisfaction. They are, almost without exception, innocent, harmless men, with a great appetite for adulation, and subject to the same weaknesses that plague ordinary mortals. They really seek to do the best. In dental journalism at least, their emoluments are moderate, very moderate indeed, consisting of an occasional free ticket to a professional dinner, and a long list of more or less stupid exchanges, through which they must periodically wade, while their labors are interminable, and they literally know no rest. Poor men; they deserve compassion and consideration, and not reproach and contumely. With the greatest propriety might they print at the head of each number of their several journals, the notice stuck up in the orchestra of a "dive" in one of the towns upon the western border of our civilization : "Don't shoot at the fiddlers. They are doing the best they can."

